# MRI-based Alzheimer’s disease classification using Vision Transformer and time-series transformer: A step-by-step guide

**DOI:** 10.1016/j.simpa.2025.100771

**Published:** 2025-06-10

**Authors:** Sait Alp, Sara Akan, Taymaz Akan, Mohammad Alfrad Nobel Bhuiyan

**Affiliations:** a Department of Artificial Intelligence Engineering, Trabzon University, Trabzon, 61335, Turkey; b Department of Computer Engineering, Faculty of Engineering, Istanbul Galata University, Istanbul, Turkiye; c Department of Medicine, LSU Health Shreveport, Shreveport, LA, USA; d Department of Software Engineering, Faculty of Engineering, Istanbul Topkapı, University, Istanbul, Turkiye

**Keywords:** Alzheimer’s disease, MRI, Transfer learning, Sequence classification, Vision transformer

## Abstract

This study introduces a reproducible pipeline for classifying Alzheimer’s Disease from structural brain MRI utilizing a joint transformer architecture that integrates Vision Transformer and Time-Series Transformer models. The proposed framework uses pre-trained ViT for feature extraction from 2D slices of MRI volumes, followed by sequential modeling with a transformer-based classifier to capture inter-slice dependencies. The method is evaluated on the ADNI dataset, involving both binary (AD vs. NC) and multiclass (AD, MCI, NC) classification tasks across axial, sagittal, and coronal planes.

**Table:** Code metadata

Current code version	*v1.0*
Permanent link to code/repository used for this code version	https://github.com/SoftwareImpacts/SIMPAC-2025-98
Permanent link to reproducible capsule	
Legal code license	*MIT License*
Code versioning system used	*git*
Software code languages, tools and services used	*Python*
Compilation requirements, operating environments and dependencies	TensorFlow, Keras, Sklearn
If available, link to developer documentation/manual	*N/A*
Support email for questions	nobel.bhuiyan@lsuhs.edu

## 1. Introduction

Alzheimer’s disease (AD) is a brain disorder characterized by the accumulation of abnormal protein deposits called plaques and tangles, which lead to the death of nerve cells and the degeneration of brain tissue [1,2]. The disease is categorized into three stages: preclinical, mild cognitive impairment (MCI), and dementia [3,4]. Early detection and differentiation of MCI have become crucial for successful disease treatment and management, as well as to slow disease progression and improve quality of life for those with AD. Advancements in computer-aided diagnosis (CAD) systems based on neuroimaging data tools have improved classification. Traditional approaches to image analysis employ a four-stage pipeline of pre-processing, segmentation, feature extraction, and classification [5–9]. Deep learning algorithms have an advantage over conventionally based methods because they require little or no image pre-processing, resulting in a more objective and less biased process. CNN-based architectures are widely used for medical image analysis, but they cannot be used to construct deep models due to their high computational requirements [10].

Transformer architecture, which dominates natural language processing, has been limited in medical imaging. However, Vision Transformer (ViT) has gained popularity due to its impressive results in various medical imaging tasks, including image classification, object detection, and semantic segmentation [11]. ViT uses a multi-headed self-attention mechanism to capture long-range dependencies, extracting features across the entire image without degrading image resolution, preventing spatial loss from information skipping. ViT is based on the concept of Transformers from natural language processing applied to medical images, using a standard Transformer architecture to process MRI images instead of text. The joint transformer handles long-range dependencies, avoids recursion, and allows parallel computation to reduce training time and avoid performance drops due to long-range dependencies.

## 2. Method overview

We present a two-stage deep learning approach for classifying Alzheimer’s disease (AD) using 3D T1-weighted MRI scans. Our pipeline first extracts features from 2D slices using a Vision Transformer (ViT) and then models inter-slice relationships with a transformer-based sequence classifier.

### MRI Preprocessing:

T1-weighted MRI volumes were preprocessed using SPM12 and CAT12 toolboxes. Images were standardized to MNI space, skull-stripped, and resampled across axial, coronal, and sagittal planes.

### 2D ViT Feature Extraction:

To leverage pre-trained 2D models (e.g., ImageNet21K), each 3D MRI was split into 2D slices. Each slice was processed independently: sizes were standardized and resized to 224 × 224 pixels, divided into 14 × 14 patches (16 × 16 pixels). These patches were fed into a Vision Transformer to extract high-dimensional feature vectors from each slice.

### Time-Series Transformer for Sequence Classification:

The sequential features obtained from ViT were then input into a time-series transformer model to preserve spatial continuity and inter-slice dependencies. This model captured long-range dependencies using self-attention without requiring additional embedding layers.

## 3. Software description

To begin using the ViT-TST classification pipeline, it is essential to prepare the MRI data correctly. The overall data preparation workflow includes preprocessing 3D MRI scans, extracting the central slices, saving them into patient-specific folders, resizing, and later extracting features with Vision Transformer (ViT). This section details both the conceptual workflow and the operational steps using the provided scripts. The classification pipeline is based on three key Python scripts that must be executed in sequence:

1. extract_save_features_ViT.py – extracts and saves features from preprocessed MRI slices using a Vision Transformer.
2. splitDatasetFeatureK_Fold.py – creates 10-fold cross-validation datasets from the saved features.
3. MRI_timeseriClassificationTransformer_keras_Kfold.py – trains and evaluates a time-series transformer model for classification.

This section outlines how to prepare the data to be compatible with these scripts. The pipeline starts with preprocessing MRI scans, saving central slices, and extracting features.

### 3.1. Data preparation

- **Registration & Skull-Stripping**
  - Use CAT12 toolbox in SPM12 (MATLAB)
  - Standardize all scans to MNI space
  - Perform skull-stripping to eliminate non-brain tissue
- **Slice Extraction**
  - For each subject, create a separate folder named with the patient ID. (e.g., ./prepared_data/PATIENT_ID/).
  - Slice the 3D volume along the chosen plane (axial, coronal, or sagittal).
  - From the full stack of slices, select the central 50 slices.
  - Resize each slice to 224 × 224 pixels to match the input resolution of pre-trained ViT models
  - Save each slice as a separate 2D image file (e.g., PNG or JPEG) inside the patient’s folder.

**Fig. 1.**
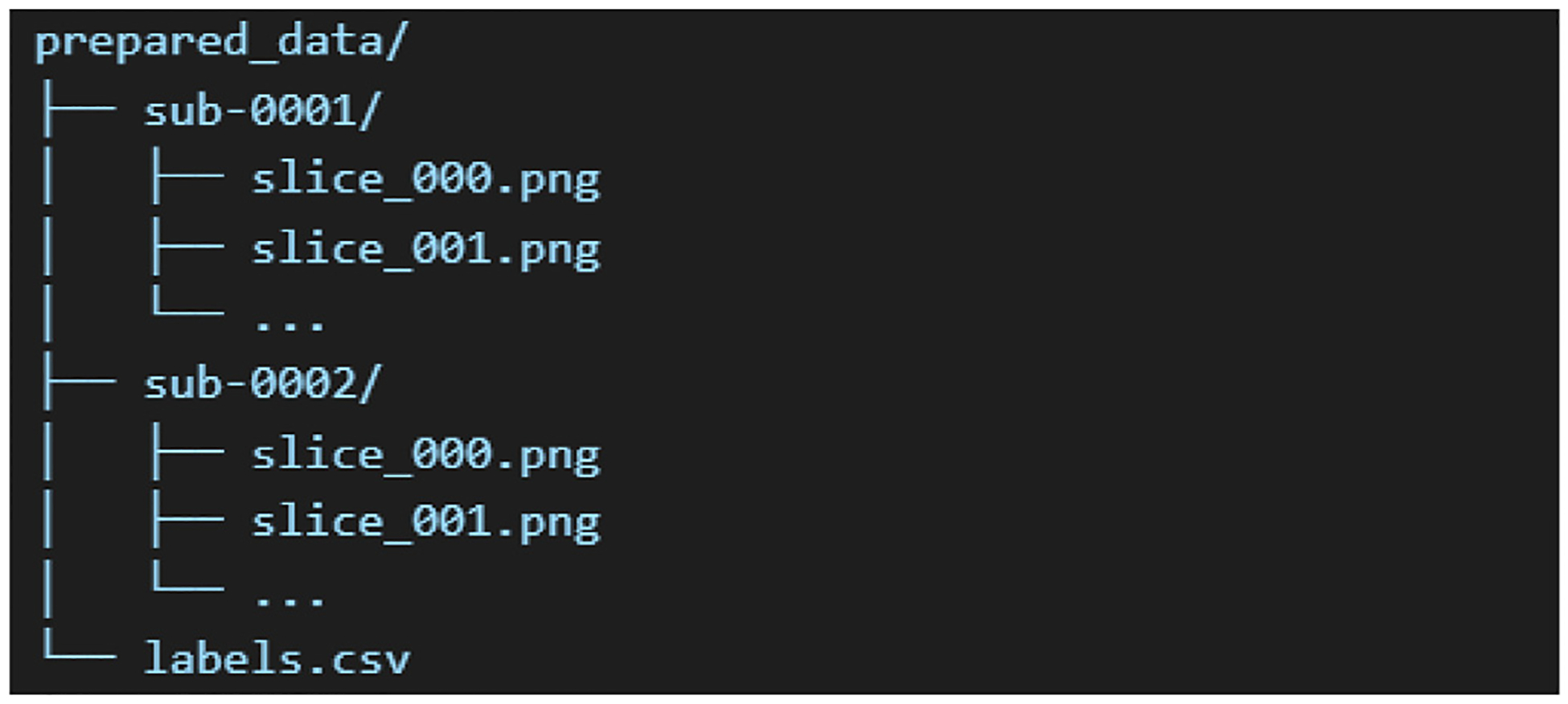
Example directory structure used for organizing MRI data and extracted features in the proposed pipeline. Each folder corresponds to a single subject and contains the central 50 slices in axial, coronal, and sagittal plane.

This structured preparation ensures consistency across subjects and enables downstream processing. An example directory structure is shown in Fig. 1.

### 3.2. Feature extraction using ViT

- Use extract_save_features_ViT.py to load the 50 slices from each folder, and feed them through a pre-trained ViT model.
- The extracted feature sequence (one vector per slice) is saved as a “.npy” file inside the same folder or a new output directory. The features for each patient are saved as:
  - scan_data.npy: 2D array (number of slices × feature dimension)
  - scan_labels.npy: single label for diagnosis (NC, MCI, or AD)

This command will process all preprocessed scans, extract ViT features from the central 50 axial slices, and save the outputs into the ./features_axial/ directory. Each patient’s extracted features are saved as .npy files corresponding to the data, labels, and masks, organized in a flat file structure rather than subdirectories per patient.

### 3.3. Generate 10-fold cross-validation dataset

This command will load all feature and label files from “./features_output/”, split them into 10 folds, and save the partitioned files in “./kfold_splits/”. Once the ViT features have been extracted and saved, the next step is to organize the dataset for training and evaluation. This is done using splitDatasetFeatureK_Fold.py, which partitions the data into ten stratified folds for cross-validation. The script reads the .npy files (scan_data.npy, scan_labels.npy) generated in the feature extraction step. It uses scikit-learn’s StratifiedShuffleSplit to ensure that class distributions are maintained in each fold. For each of the 10 folds, the data is further split into training and validation sets (typically 80/20 split), in addition to the test set. For each fold i = 1 to 10, the script will generate:

- traindata_Foldi.npy, trainlbl_Foldi.npy
- validdata_Foldi.npy, validlbl_Foldi.npy
- testdata_Foldi.npy, testlbl_Foldi.npy

### 3.4. Sequence modeling using time-series transformer

The final step in the pipeline is to train and evaluate a transformer-based sequence classification model using the previously generated folds. This is performed with the script MRI_timeseriClassificationTransformer_keras_Kfold.py, which loads the extracted features and corresponding labels for each fold, trains a model, and evaluates its performance.

This script is built using TensorFlow/Keras and implements a time-series transformer architecture tailored for sequence data. The model receives a sequence of ViT feature vectors (one per slice) and learns to classify the entire MRI volume. The transformer architecture includes positional encodings, multi-head self-attention layers, and dense classification heads.

#### 3.4.1. Steps performed

1. For each fold i from 1 to 10:
  o Load traindata_Foldi.npy, trainlbl_Foldi.npy
  p Load validdata_Foldi.npy, validlbl_Foldi.npy
  q Load testdata_Foldi.npy, testlbl_Foldi.npy
2. Build the Transformer model using Keras layers:
  o Input shape: (50, feature_dim)
  p Positional encoding layer
  q Multiple Transformer blocks (multi-head attention + feed-forward layers)
  r Global average pooling or attention-based pooling
  s Dense output layer with softmax activation
3. Compile and train the model using categorical cross-entropy loss.
4. Evaluate the model on the test set and compute metrics Evaluation is performed independently for each of the 10 folds. For each fold, the trained model is tested using the hold-out test set, and the classification performance is assessed using standard metrics (Accuracy, Precision, Recall, and F1-score). hese scores are computed using the sklearn.metrics package. After all folds are evaluated, the average score across all folds is calculated for each metric. This provides a reliable estimate of the model’s generalization performance. The output of the script consists of classification metrics computed independently for each of the 10 folds. After all folds are evaluated, the average values of these metrics across the 10 folds are calculated and reported. Optionally, confusion matrices can be generated to show detailed class-wise performance.

## 4 Impact overview

Regarding the experimental results presented in [10], the Joint Transformer architecture developed in this work significantly enhances the classification accuracy of AD using brain MRI volume. By jointly leveraging ViT for spatial feature extraction and Time-Series Transformers for capturing slice-wise dependencies, the proposed model surpasses conventional convolutional approaches. Compared to standard CNN-based methods, our model demonstrated a balanced accuracy of 86.8% and a F1-score of 86.6%, outperforming established baselines such as ResNet50 and DenseNet121. Furthermore, the model maintained stable performance across 10-fold cross-validation, confirming its robustness and reproducibility. Previous methods often struggled to generalize across different MRI acquisition protocols, while our transformer-based method exhibited better adaptability without extensive fine-tuning, particularly important for clinical application scenarios.

The complete source code is made publicly available on GitHub, enabling researchers and developers to easily access, reproduce, and extend the system. The software is modular and scalable, allowing integration with different MRI preprocessing pipelines and classification workflows. Key aspects that maximize the impact of our software include:

1. Efficient transformer-based design capturing both spatial and sequential MRI features without reliance on convolutional operations.
2. Scalable architecture facilitating adaptation to different MRI resolutions and datasets.

Besides the success of the joint transformer model for MRI classification, the provided software package plays a critical role in enabling reproducible machine learning research in medical imaging. By improving classification performance while maintaining generalization across varying data distributions, this work contributes a reliable and extensible foundation for future research in neurodegenerative disease analysis.

## References

[1] Hazarika RA, Kandar D, Maji AK, An experimental analysis of different Deep Learning based Models for Alzheimer’s Disease classification using Brain Magnetic Resonance Images, J. King Saud Univ. - Comput. Inf. Sci 34 (10) (2022) 8576–8598, 10.1016/J.JKSUCI.2021.09.003.

[2] Jain R, Jain N, Aggarwal A, Hemanth DJ, Convolutional neural network based Alzheimer’s disease classification from magnetic resonance brain images, Cogn. Syst. Res 57 (2019) 147–159, 10.1016/J.COGSYS.2018.12.015.

[3] Blennow K, Henrik Z, Fagan AM, Fluid biomarkers in Alzheimer disease, 2012, 10.1101/cshperspect.a006221. PMC3426814 22951438

[4] Khojaste-Sarakhsi M, Haghighi SS, Ghomi SMTF, Marchiori E, Deep learning for Alzheimer’s disease diagnosis: A survey, Artif. Intell. Med 130 (2022) 102332, 10.1016/J.ARTMED.2022.102332. 35809971

[5] Yue L, Gong X, Li J, Ji H, Li M, Nandi AK, Hierarchical feature extraction for early Alzheimer’s disease diagnosis, IEEE Access 7 (2019) 93752–93760, 10.1109/ACCESS.2019.2926288.

[6] Silva IRR, Silva GSL, De Souza RG, Dos Santos WP, De Fagundes RAA, Model based on deep feature extraction for diagnosis of Alzheimer’s disease, in: Proceedings of the International Joint Conference on Neural Networks, 2019, 10.1109/IJCNN.2019.8852138.

[7] Zhang F, Li Z, Zhang B, Du H, Wang B, Zhang X, Multi-modal deep learning model for auxiliary diagnosis of Alzheimer’s disease, Neurocomputing 361 (2019) 185–195, 10.1016/J.NEUCOM.2019.04.093.

[8] Zhao B, Lu H, Chen S, Liu J, Wu D, Convolutional neural networks for time series classification, J. Syst. Eng. Electron 28 (1) (2017) 162–169, 10.21629/JSEE.2017.01.18.

[9] Wen Q, Zhou T, Zhang C, Chen W, Ma Z, Yan J, Sun L, Transformers in time series: A survey, 2022, 10.48550/arxiv.2202.07125.

[10] Alp S, Akan T, Bhuiyan MS, Disbrow EA, Conrad SA, Vanchiere JA, Kevil CG, Joint transformer architecture in brain 3D MRI classification: its application in Alzheimer’s disease classification, Sci. Rep 14 (1) (2024) 8996. 38637671 10.1038/s41598-024-59578-3 PMC11026447

[11] Dosovitskiy A, Beyer L, Kolesnikov A, Weissenborn D, Zhai X, Unterthiner T, Dehghani M, Heigold G, Gelly S, Uszkoreit J, An image is worth 16 × 16 words: Transformers for image recognition at scale. Retrieved from https://github.com/.

